# Myocarditis After mRNA Vaccination: A Metabolic–Innate Immune Cascade Centered on Lipid Nanoparticles

**DOI:** 10.1155/mi/8991922

**Published:** 2026-06-09

**Authors:** Kangchan Choi, Seungchan Choi, Daeho Joe, Yie-sung Seo, Jeehye Ham, Hannah Chung, Yousef Ramadan, Yong Serk Park

**Affiliations:** ^1^ Regeneration Medicine Research Center, Yonsei University Wonju College of Medicine, Wonju, 26426, Republic of Korea, yonsei.ac.kr; ^2^ School of Medicine, Trinity Medical Sciences University, Ratho Mill, VC0272, Saint Vincent and the Grenadines; ^3^ Bio-Pharmaceutical Convergence Major, School of Pharmacy, Sungkyunkwan University, Suwon, 16419, Republic of Korea, skku.edu; ^4^ Biomedical Laboratory Science, College of Health Science, Yonsei University, Wonju, 26493, Republic of Korea, yonsei.ac.kr

**Keywords:** fatty-acid oxidation, innate immunity, lipid nanoparticles, mRNA vaccines, myocarditis, NLRP3

## Abstract

mRNA vaccine‐associated myocarditis is a rare but clinically important adverse event whose pathogenesis remains incompletely understood. Initial hypotheses focused primarily on the spike protein antigen, with growing preclinical evidence implicating the lipid nanoparticle (LNP) delivery system as an additional and potentially important contributor to myocardial inflammation. Here, we propose a multi‐hit model that integrates LNP‐driven mechanisms as a central pathogenic axis, initiated by the systemic distribution and accumulation of LNPs in the heart. While the mRNA payload is cleared within days, the synthetic ionizable lipids ‐ALC‐0315 (BNT162b2) and SM‐102 (mRNA‐1273) persist significantly longer than the mRNA payload itself. These two lipids differ in biodegradability and pharmacokinetic distinctions, together with differences in lipid dose and formulation, they may contribute to the divergent myocarditis rates observed between the two vaccine products. In this suggesting review, the first hit” involves the disruption of myocardial energy metabolism by these lipids, which can integrate into cellular membranes and impair mitochondrial fatty acid oxidation. This is compounded by a second hit of direct innate immune activation, preclinical studies demonstrate that LNPs engage pattern‐recognition receptors (PRRs) like toll‐like receptors (TLRs) and the NLRP3 inflammasome, leading to the release of pro‐inflammatory cytokines such as IL‐1*β* and IL‐18. Inflammation is then amplified via Damage‐associated molecular patterns (DAMPs) released from stressed cardiomyocytes. The clinical outcome—ranging from self‐limited mild myocarditis to fulminant disease with diverse histopathological patterns—is likely shaped by host susceptibility factors, including sex hormones, genetic predisposition, and prior immune priming, that modulate the intensity of this pathogenic cascade.

## 1. The Clinical Problem

Myocarditis (inflammation of the heart muscle), pericarditis (inflammation of the pericardial sac), and myopericarditis (concurrent involvement of both) have distinct definitions, mechanisms, and clinical implications. We used the term “vaccine‐associated myocarditis” in general to encompass cases with predominant myocardial involvement. The monumental success of messenger RNA (mRNA) vaccines in combating the COVID‐19 pandemic [1, 2] has been tempered by the identification of a rare but important safety signal: acute myocarditis and pericarditis. This phenomenon displays a distinct epidemiological pattern, occurring predominantly in male adolescents and young adults under 30 [3–6], typically within a week following a second dose of an mRNA vaccine, particularly the mRNA‐1273 (Moderna) vaccine [7, 8]. A large population‐based study in Ontario, Canada, reported an incidence of myocarditis as high as 299.5 cases per million second doses of mRNA‐1273 in males aged 18–24 [9]. This rate is substantially higher than that observed after the BNT162b2 (Pfizer‐BioNTech) vaccine (59.2 cases per million) [9] or in other demographic groups, a finding consistent across multiple international cohort studies [10, 11]. On the other hand, it is increasingly recognized that vaccine‐associated myocarditis encompasses a broader clinical spectrum. Older patients, those with balanced sex distribution, and cases with fulminant presentation and diverse histopathological patterns (including eosinophilic and mixed inflammatory infiltrates) have been documented, indicating that a single pathogenic mechanism is unlikely to account for all cases. The causal relationship between mRNA COVID‐19 vaccines and myocarditis has been formally established [12]. This pronounced risk stratification by vaccine type, age, and sex strongly suggests the existence of a sophisticated pathophysiological mechanism that extends beyond simple, nonspecific inflammation (Table 1).

**Table 1:** Summary of key epidemiological, histopathological, and regulatory evidence on mRNA vaccine‐associated myocarditis.

Design	Population	BNT162b2 incidence ^∗^	mRNA‐1273 incidence ^∗^	Key finding
US VAERS [5]	US (All ages)	52.4	56.3	Highest incidence in young males after dose 2 Predominantly mild clinical course
Population cohort [9]	Ontario, Canada (≥18 years)	59.2	299.5	~5× higher risk with mRNA‐1273 vs. BNT162b2 in young males
Population‐based cohort [3]	Nordic countries (23M residents)	IRR: 5.31 (95% CI, 3.68–7.68)	IRR: 13.83 (95% CI, 8.08–23.68)	Excess events per 100,000 5.55 (BNT) vs. 18.39 (mRNA‐1273)
Head–to–head comparison [13]	US VSD (18–39 years)	24.1	33.0	Head–to–head RR: 1.61 (95% CI 1.02–2.54) for mRNA‐1273 vs. BNT162b2
COMBAT study [14]	Japan (40 biopsy cases)	—	—	47.5% mild (no cardiomyocyte injury) 52.5% with injury (lymphocytic, eosinophilic, mixed) 1.4% of injury group developed fulminant myocarditis
Comprehensive evidence review [12]	All available data (Epi and mechanistic)	Causal relationship established	Causal relationship established; risk likely greater than BNT162b2	Causal relationship established for both vaccines Mechanistic pathways identified (TLR4/inflammasome/IL‐1*β*)
Post‐marketing surveillance (BEST system, FDA 2025)	Commercial claims (6 months–64 years)	Combined: ~8 overall, ~27 in males 12–24 years	Combined with BNT162b2 (not reported separately)	Persistence of abnormal CMR findings at median 5‐month follow‐up

*Note:* Incidence rates represent reported cases per million doses in the highest‐risk demographic (typically males aged 12–24 years after dose 2) unless otherwise noted. —: data not separately reported by vaccine product.

Abbreviations: BEST, biologics effectiveness and safety system; CI, confidence interval; CMR, cardiac magnetic resonance; IRR, incidence rate ratio; RR, rate ratio; VAERS, vaccine adverse event reporting system; VSD, vaccine safety datalink.

^∗^Incidence represented as cases per 1 million doses (males aged 18–24 years, dose 2), unless stated otherwise.

While initial hypotheses centered on the spike protein antigen itself [15]—implicating direct toxicity or autoimmunity via molecular mimicry [16]—these theories do not fully account for the platform‐specific nature (i.e., rarity with adenoviral vector vaccines) [17] or the rapid onset of the condition. However, these spike‐centered models do not explain key epidemiological features of the condition such as platform‐specific incidence patterns, dose‐dependent risk differentials between BNT162b2 and mRNA‐1273, and the rapid onset within days of vaccination.

**Figure 1:**
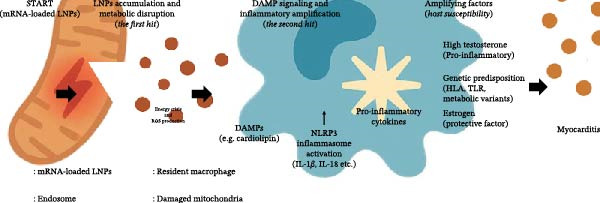
Proposed multi‐hit pathophysiological model of mRNA vaccine‐associated myocarditis. The model integrates several key events. LNP accumulation and metabolic disruption: Intramuscularly injected LNPs distribute to the heart, where they accumulate in endothelial cells and cardiomyocytes, disrupting fatty acid metabolism and causing mitochondrial injury. DAMP signaling and inflammatory amplification): Damaged mitochondria release DAMPs, such as cardiolipin, which, along with the LNP itself, activate innate immune sensors (TLRs, NLRP3), leading to a surge in pro‐inflammatory cytokines. Amplifying factors: This cascade is modulated by host susceptibility factors, including genetics (e.g., HLA type, immune/metabolic gene polymorphisms) and hormones (e.g., testosterone), which determine the final clinical outcome. This figure illustrates the LNP‐centered pathway that is the focus of this review. It should be noted that there are additional mechanisms that might operate in parallel or predominant in some patient subsets (Table 2).

Accordingly, we posit a new paradigm that shifts the focus to the lipid nanoparticle (LNP) delivery system as a central instigator of myocardial injury. We propose a “multi‐hit” model wherein the direct metabolic toxicity and innate immune activation induced by the LNP’s synthetic lipids converge with host‐specific susceptibility factors to trigger clinical myocarditis (Figure 1, Table 1).

**Table 2:** Proposed mapping of histopathological phenotypes to potential pathogenic mechanisms in vaccine‐associated myocarditis.

Histopathological phenotype	Predominant infiltrate	Clinical profile	Predominant proposed mechanism	Severity
Mild lymphocytic without cardiomyocyte injury	Lymphocytes, interstitial edema	Young males, chest pain, preserved LVEF	LNP‐driven innate activation (TLR/NLRP3) ± subclinical metabolic stress	Mild, self‐limited
Lymphocytic with cardiomyocyte injury	T‐cells, macrophages	Variable age and sex	Autoimmune/adaptive response (molecular mimicry, HLA‐restricted) ± LNP‐initiated cascade	Moderate to severe
Eosinophilic	Eosinophils, macrophages	Variable; may include older patients	Hypersensitivity reaction to LNP components or spike protein	Moderate to fulminant
Mixed (lymphocytic + eosinophilic)	Lymphocytes + eosinophils	Variable	Overlap of innate/adaptive and hypersensitivity pathways	Moderate to fulminant
Giant cell (rare)	Giant cells, lymphocytes, eosinophils	Severe presentation	Severe autoimmune‐mediated; may require aggressive immunosuppression	Fulminant

*Note:* Phenotype classifications are derived primarily from the COMBAT study and supplemental biopsy series. Note that the proposed mechanisms are conceptual frameworks intended to highlight the pathophysiological heterogeneity of the condition, rather than established causal pathways.

Abbreviations: HLA, human leukocyte antigen; LNP, lipid nanoparticle; LVEF, left ventricular ejection fraction; NLRP3, NLR family pyrin domain‐containing 3; TLR, toll‐like receptor.

## 2. Pathophysiological Mechanisms

A systematic literature search was conducted across PubMed, Scopus, and Web of Science using the following terms: “mRNA vaccine myocarditis,” “lipid nanoparticle in cardiac,” “LNP innate immunity,” “ionizable lipid inflammasome,” and “vaccine‐associated myocarditis mechanism.”

### 2.1. Biokinetics and Cardiac Persistence of LNPs

**Figure 2:**
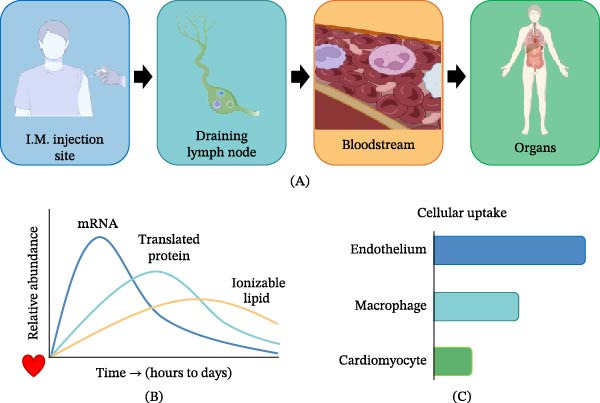
Spatiotemporal biodistribution and persistence of mRNA–LNP components after intramuscular dosing (conceptual). (A) IM depot drains to lymph nodes and blood, producing transient systemic exposure with trace heart delivery (peak ~2–4 h). (B) Qualitative time‐course at the heart contrasts rapid clearance of mRNA and translated protein with the longer‐tail persistence of ionizable lipids. The curves are schematic and do not represent measured concentrations at clinical vaccine doses. (C) Within cardiac tissue, uptake is enriched in endothelium and resident macrophages relative to cardiomyocytes. Curves/bars are schematic (not measured values); organ‐ and chemistry‐specific kinetics are simplified.

Following intramuscular injection, a fraction of LNPs enters systemic circulation and distributes to distal organs, including the heart [18, 19] (Figure 2A). Quantitative animal studies demonstrate that LNPs reach peak concentration in cardiac tissue within 2–4 h post‐injection and persist with a half‐life of 25–48 h. However, we should note that the absolute quantity reaching the heart is small; systematic analyses of nanoparticle biodistribution indicate that cardiac accumulation typically represents less than 2% of the injected dose per gram of tissue after intravenous administration, and even smaller fraction is expected after intramuscular injection [20]. Nevertheless, some LNP components can be detected in the heart for at least 72 h (Figure 2B), and an even trace‐level exposure of the metabolically active myocardium to bioactive synthetic lipids could be biologically significant, considering the heart’s continuous energy demands and limited regenerative capacity.

**Figure 3:**
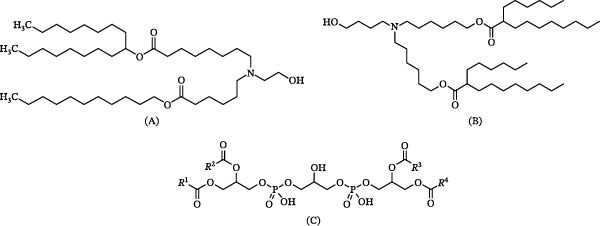
Chemical structures of ionizable lipids and cardiolipin. (A) SM‐102, the ionizable lipid used in the Moderna (mRNA‐1273) vaccine. (B) ALC‐0315, the ionizable lipid used in the Pfizer‐BioNTech (BNT162b2) vaccine. (C) Cardiolipin, a key phospholipid of the inner mitochondrial membrane. The synthetic ionizable lipids (A, B) possess a tertiary amine headgroup (cationic at endosomal pH) and two acyl chains. In contrast, cardiolipin (C) has a diphosphate headgroup (anionic at physiological pH, charge ≈ −2) and four acyl chains. These fundamental differences in charge, headgroup chemistry, and geometry make a direct molecular mimicry between them unlikely.

The persistence of these synthetic lipids is a key factor. The two vaccines utilize different ionizable lipids: BNT162b2 uses ALC‐0315, which lacks an easily hydrolyzable bond and is cleared slowly [21, 22], while mRNA‐1273 uses SM‐102, which contains an ester linkage designed for more rapid biodegradation [23] (Figure 3).

Paradoxically, mRNA‐1273 is associated with a higher incidence of myocarditis, despite using a more biodegradable lipid. This obvious contradiction may be reconciled by considering the substantially higher mRNA dose (100 μg vs. 30 μg for BNT162b2), corresponding to a greater total mass of ionizable lipid per injection and the higher molar ratio of ionizable lipid in the mRNA‐1273 formulation (50.0% vs. 46.3%; Table 3). Thus, the net cardiac exposure to synthetic lipids—determined by both the dose administered and the rate of clearance—may ultimately be greater with mRNA‐1273, consistent with its higher clinical risk signal.

**Table 3:** Comparison of LNP lipid composition in two mRNA vaccines.

Components	BNT162b2 (Pfizer)	mRNA‐1273 (Moderna)	Difference
Ionizable lipid	46.3 (ALC‐0315)	50.0 (SM‐102)	3.70%
PEG‐lipid	1.6 (ALC‐0159)	1.5 (DMG‐PEG_2000_)	−0.10%
Cholesterol	42.70	38.50	−4.20%
DSPC (Helper lipid)	9.40	10.00	0.60%

*Note:* Molar ratios (%) of the four main lipid components (ionizable lipid, PEG‐lipid, cholesterol, and DSPC) in the BNT162b2 (Pfizer) and mRNA‐1273 (Moderna) vaccines. The mRNA‐1273 vaccine, which is associated with a higher incidence of myocarditis, contains a higher molar ratio of ionizable lipid (50.0 vs. 46.3) and a lower ratio of cholesterol compared to the BNT162b2 vaccine. This suggests that the dose and formulation of the ionizable lipid may be critical risk factors, directly influencing the intensity of the “first hit” (metabolic disruption) in our proposed model. SM‐102: 9‐Heptadecanyl 8‐{(2‐hydroxyethyl)[6‐oxo‐6‐(undecyloxy)hexyl]amino}octanoate. ALC‐0315: [(4‐Hydroxybutyl)azanediyl]di(hexane‐6,1‐diyl) bis(2‐hexyldecanoate). ALC‐0159: 2‐[(polyethylene glycol)‐2000]‐N,N‐ditetradecylacetamide. DSPC: 1,2‐distearoyl‐sn‐glycero‐3‐phosphocholine. DMG‐PEG_2000_: 1,2‐dimyristoyl‐rac‐glycero‐3‐methoxypolyethylene glycol‐2000.

Furthermore, single‐cell analyses reveal that within the heart, LNPs are preferentially taken up not by cardiomyocytes, but by vascular endothelial cells and resident macrophages [24] (Figure 2C). The preferential uptake of LNPs by vascular endothelial cells and resident macrophages suggests that the initial injury cascade may originate in the cardiac microvasculature and local immune milieu, rather than from direct damage to the contractile cells themselves. Such endothelial tropism is also consistent with alternative injury mechanisms, including complement‐mediated endothelial activation or hypersensitivity‐type reactions, which may operate in parallel with or independently of the metabolic pathway described below.

### 2.2. The First Hit: Metabolic Disruption of the Myocardium by Synthetic Lipids

The myocardium is a metabolic omnivore with a profound reliance on mitochondrial fatty acid β‐oxidation, which supplies 60%–90% of its ATP [25, 26]. The cationic, amphiphilic nature of the ionizable lipids is known to allow them to integrate into cellular and organellar membranes in general. Based on the established pharmacology of structurally analogous cationic amphiphilic drugs, it is plausible that these lipids could interfere with this finely tuned metabolic machinery, although direct demonstration of this process in the human myocardium at vaccine‐relevant concentrations remains to be established [27–31].

• Interference with fatty acid metabolism: These synthetic lipids may disrupt mitochondrial fuel supply by interfering with key enzymes. A plausible target is carnitine palmitoyltransferase‐1 (CPT‐1), the rate‐limiting enzyme for fatty acid entry into the mitochondria [32, 33]. While direct inhibition of CPT‐1 by vaccine‐derived ionizable lipids has not yet been demonstrated, structurally similar cationic amphiphilic compounds are known to accumulate in mitochondrial membranes and impair fatty acid transport. Competitive inhibition of this gateway could precipitate an “energy crisis” by blocking the heart’s primary fuel source. Furthermore, disruption of lipid‐regulating pathways like SIRT1‐SCD1 can alter cell membrane composition, inducing endoplasmic reticulum (ER) stress [34].
• Consequences of metabolic stress: This metabolic perturbation leads to mitochondrial dysfunction, reduced ATP production, and a surge in reactive oxygen species (ROS) [35, 36]. In vitro studies on isolated rat cardiomyocytes have demonstrated that the two vaccine formulations induce distinct functional disturbances: mRNA‐1273 LNP caused arrhythmic, irregular contractions linked to calcium‐handling defects, whereas BNT162b2 LNP caused hypercontractility via protein kinase A activation [37, 38]. These vaccine‐specific functional signatures are notable and suggest that the formulation differences between the two products have direct biological consequences at the cellular level. However, several important limitations must be acknowledged. First, the concentrations used in these in vitro experiments (up to 3.3 μg mRNA/mL) may exceed the transient local concentrations achieved in cardiac tissue following standard intramuscular vaccination, where only a small fraction of the injected dose reaches the heart (Section 2.1). Second, because unloaded or control‐mRNA‐loaded LNPs were not available for comparison, the observed effects cannot be unequivocally attributed to the LNP component alone; contributions from the translated spike protein remain possible. Notwithstanding these caveats, the demonstration that each vaccine formulation produces a pharmacologically distinct cardiomyocyte phenotype supports the hypothesis that LNP composition contributes to the divergent clinical risk profiles of BNT162b2 and mRNA‐1273.

### 2.3. The Second Hit: Innate Immune Activation and DAMP‐Mediated Amplification

The LNP is not a passive vehicle; accumulating evidence indicates that it functions as an innate immune adjuvant, capable of activating inflammatory pathways independently of the mRNA [39–42]. Notably, recent studies using empty LNPs (devoid of mRNA) have demonstrated that the LNP carrier itself elicits NF‐κB and IRF signaling at levels comparable to mRNA‐loaded LNPs, confirming a substantial contribution of the lipid components to innate immune activation. This activation appears to occur through two synergistic pathways:

• Direct inflammasome activation: The ionizable lipids within the LNP are recognized by multiple pattern‐recognition receptors (PRRs) [43, 44]. They are sensed by endosomal toll‐like receptors (TLRs) and, crucially, act as a powerful trigger for the NLRP3 inflammasome in pre‐clinical models [40, 45–47]. Mechanistically, the amine headgroup of ionizable lipids plays a key role in this response by binding to TLR4 and CD1d, as demonstrated for several lipid species. Additionally, the potency of inflammasome activation varies by lipid type: SM‐102‐containing LNPs, for instance, provoke a much stronger IL‐1β release than LNPs made with other ionizable lipids such as MC3. This differential inflammasome activation by SM‐102 may provide a mechanistic link to the higher incidence of myocarditis observed with mRNA‐1273 (Section 2.1). High concentrations of ionizable lipids at the cellular level can destabilize lysosomal membranes, causing rupture [46, 48, 49]—a danger signal that leads to the assembly of the NLRP3 inflammasome and activation of caspase‐1 [50–52]. Activated caspase‐1 then cleaves pro‐IL‐1*β* and pro‐IL‐18 into their highly inflammatory, active forms [53]. Importantly, the lysosomal rupture in these studies generally occurred at lipid concentrations exceeding those estimated to reach the heart after standard intramuscular vaccination; whether this mechanism operates at the trace concentrations present in cardiac tissue remains an open question.
• DAMP‐mediated amplification loop: The initial metabolic injury (the “first hit") would be expected to cause stressed or dying cardiomyocytes to release endogenous danger signals known as damage‐associated molecular patterns (DAMPs). A key DAMP relevant to this context is cardiolipin, a unique phospholipid of the inner mitochondrial membrane [54–56]. When externalized due to mitochondrial stress, cardiolipin itself becomes a potent trigger for the NLRP3 inflammasome [57, 58]. This could create a vicious feed‐forward loop: LNP‐induced metabolic stress causes cardiolipin release, which in turn amplifies inflammasome‐driven inflammation, leading to more cell death and further DAMP release. While this amplification cascade remains a theoretical construct that has not been directly demonstrated in the myocardium in vivo, indirect clinical evidence is consistent with inflammasome involvement [59, 60]. Specifically, elevated levels of IL‐18 observed in patients with vaccine‐associated myocarditis are consistent with an inflammasome‐centric mechanism [15, 61, 62]. However, since IL‐18 can also be elevated through other inflammatory pathways, these findings support but do not conclusively prove the proposed LNP‐inflammasome‐DAMP cascade.

## 3. The Decisive Factors

The predisposition of certain individuals, particularly young males, to develop myocarditis suggests that the metabolic and innate immune perturbations described above (Section 2.2 and 2.3) are not sufficient on their own. Instead, host‐specific factors act as critical modulators that either amplify or dampen the pathogenic cascade. These underlying traits dictate the final clinical severity, ranging from silent myocardial inflammation to fulminant myocarditis. Grasping these factors is critical, especially since the clinical spectrum goes beyond the classic presentation of a young man with a mild, resolving condition (Table 1).

• The immunomodulatory dichotomy: The striking male predominance is likely explained by the divergent effects of sex hormones on the immune system [63, 64]. Testosterone, which peaks in adolescent and young adult males, is known to skew immune responses toward a pro‐inflammatory Th1 profile, characterized by cytokines like IFN‐*γ* and IL‐2 [65]. This can amplify the innate immune response to LNPs, acting as an accelerator” for inflammation. For instance, testosterone can enhance the production of inflammatory mediators like IL‐1*β* and increase the expression of TLR4 [66]. Conversely, estrogen generally promotes anti‐inflammatory pathways [67, 68]. It enhances the function and stability of regulatory T cells (*T*_regs_)—the immune system’s peacekeepers—which suppress excessive inflammation through the release of cytokines like IL‐10 and TGF‐*β* [69]. This protective effect may explain the significantly lower incidence of myocarditis in females [70]. Age acts as another independent variable shaping the innate immune reaction to LNPs, quite apart from hormonal factors. For instance, a study of empty LNP‐driven immune responses revealed distinct generational differences: younger adults generated a vigorous maturation of dendritic cells, characterized by upregulated CD40 and substantial output of IFN‐*γ* and pro‐inflammatory cytokine output. In contrast, older adults exhibited impaired type I interferon signaling and reduced phagocytosis.
• The individual blueprint for risk: An individual’s genetic background is a critical determinant of susceptibility. Recent studies have identified associations between vaccine‐associated myocarditis and specific human leukocyte antigen (HLA) alleles (e.g., HLA‐DRB1 ^∗^04:01), implicating a role for antigen presentation in a subset of patients where autoimmune responses may be involved [71]. Beyond HLA, polymorphisms in genes encoding innate immune sensors (e.g., gain‐of‐function variants in *TLR3*, *TLR7*, or *NLRP3*) could lead to an exaggerated inflammatory response to the LNP [44]. Similarly, variants in genes involved in lipid metabolism could impair the clearance of synthetic lipids, prolonging their cardiotoxic effects [72, 73]. Furthermore, because several key immune‐regulatory genes (e.g., *FOXP3*, *CD40LG*) are located on the X‐chromosome, males may be more vulnerable to the effects of deleterious variants in these genes [74, 75]. Collectively, these genetic considerations suggest that vaccine‐associated myocarditis is a polygenic, multifactorial condition in which different combinations of host genetic variants may predispose to distinct pathogenic pathways—LNP‐driven innate activation in some, autoimmune‐mediated injury in others, and hypersensitivity reactions in yet another subset—rather than a single uniform mechanism.
• An epigenetic legacy of inflammation: The concept of “trained immunity” proposes that innate immune cells can be epigenetically reprogramed by an initial stimulus, leading to a heightened response to a secondary, unrelated trigger [76]. LNP vaccination itself has been shown to induce transient epigenetic and transcriptomic reprograming of monocytes [77–79]. It is plausible that in susceptible individuals, this creates a long‐lasting “inflammatory memory” in cardiac‐resident macrophages or even cardiomyocytes. This priming effect could mean that a subsequent dose of the vaccine, or another inflammatory event, triggers an outsized and pathogenic myocardial inflammatory reaction, potentially contributing to the higher risk observed after the second dose. However, other explanations—such as cumulative antigen exposure, anamnestic antibody responses, or repeated LNP‐mediated metabolic insults—may also play a role and are not mutually exclusive.

## 4. Clinical Presentation, Diagnosis, and Management

Myocarditis after mRNA vaccination typically presents within a week, most often 2–3 days after the second dose. Patients commonly report acute chest pain, often accompanied by dyspnea, palpitations, and fever. Yet, the clinical presentation and its severity vary considerably. The majority of cases, seen predominantly in young males, follow a mild, self‐limited course, while a subset of patients who are older and show a more balanced sex distribution present with fulminant disease requiring mechanical circulatory support.

Histopathology, when performed, reveals mixed inflammatory patterns rather than a single uniform phenotype. The COMBAT study performed by multiple agencies in Japan, consisting of 40 biopsy‐confirmed cases provided the most systematic characterization. In this cohort, 19 patients (47.5%) showed only mild lymphocytic infiltration and interstitial edema without cardiomyocyte injury, while the remaining 21 (52.5%) demonstrated cardiomyocyte injury with diverse inflammatory infiltrates: 11 lymphocytic, 7 eosinophilic, and 3 mixed (lymphocytic and eosinophilic) [14]. These histopathological patterns showed strong clinical correlates: 71.4% of patients with cardiomyocyte injury developed fulminant myocarditis, compared with none in the group without cardiomyocyte injury. A broader scoping review of 54 biopsy and autopsy specimens similarly documented histopathological diversity, with lymphocytic infiltration in 64.8%, eosinophilic infiltration in 29.6%, and rare cases of giant‐cell myocarditis [80].

Finding eosinophilic infiltration in a significant fraction of cases is critical. It may suggest a potential hypersensitivity‐type reaction to vaccine components rather than (or in addition to) the innate immune pathway described in our model. Ultimately, this reinforces the observation that multiple pathogenic mechanisms contribute to the full clinical spectrum of vaccine‐associated myocarditis.

The diagnosis is based on a combination of clinical symptoms and the following findings:

1. Laboratory tests: Elevation of cardiac troponin I or T, a sensitive marker of myocyte injury, is a nearly universal finding.
2. Electrocardiogram (ECG): Nonspecific changes such as ST‐segment elevation or depression and T‐wave inversions may be present.
3. Cardiac magnetic resonance (CMR): This is the most important non‐invasive diagnostic tool. Myocardial edema on T2‐weighted imaging reflects acute inflammation, while late gadolinium enhancement indicates myocyte necrosis and/or fibrosis.
4. Endomyocardial biopsy (EMB): EMB is not routinely reported but provides definitive histopathological characterization and is recommended in cases of fulminant myocarditis or when the diagnosis is uncertain [81]. As demonstrated in the COMBAT study, EMB can reveal clinically meaningful histopathological subtypes that influence prognosis and may inform management decisions.

### 4.1. Management and Prognosis

In the majority of cases, particularly those presenting as mild myocarditis in young males with preserved ventricular function, patients respond well to the treatment. Restriction of physical activity during the acute phase is a cornerstone of management. For symptomatic relief, nonsteroidal anti‐inflammatory drugs (NSAIDs) or colchicine may be used. In severe cases with hemodynamic instability or heart failure, immunomodulatory therapies such as corticosteroids, intravenous immunoglobulin (IVIG), or targeted agents like NLRP3 inhibitors or IL‐1 receptor antagonists (e.g., anakinra) may be considered. For fulminant myocarditis—which the COMBAT study documented in 71.4% of patients with histological cardiomyocyte injury—mechanical circulatory support, including extracorporeal membrane oxygenation (ECMO), may be required, and early referral to an advanced heart failure center is essential [82]. Although short‐term clinical recovery is the norm, emerging longitudinal data suggests that myocardial injury may not fully resolve in all patients. The MACiV multicenter study, which followed ~300 patients with vaccine‐associated myocarditis, found that persistence of abnormal CMR findings—including late gadolinium enhancement indicative of myocardial scarring—was common at a median follow‐up of ~5 months [83]. The clinical and prognostic significance of these persistent imaging abnormalities remains under active investigation, and long‐term studies mandated by the FDA are currently underway. These findings reinforce the importance of continued cardiac surveillance in the affected patients.

## 5. Conclusions and Future Directions

The pathogenesis of mRNA vaccine‐associated myocarditis is likely multifactorial, and a comprehensive understanding requires a paradigm shift away from the spike protein antigen alone. The pre‐clinical and clinical evidence here points toward a “metabo‐immune” pathway, where the synthetic LNP delivery system may play a central role in initiating myocardial inflammation through direct metabolic perturbation and potent innate immune activation.

This framework has significant implications. First, it highlights the need for research on improving the safety profile of LNP components. Second, it suggests potential therapeutic interventions targeting innate immune pathways (e.g., NLRP3 inhibitors) or supporting the myocardial metabolism. Third, the histopathological heterogeneity documented by biopsy studies (Table 2) implies that different patients may benefit from different therapeutic approaches—anti‐inflammatory strategies for innate‐driven cases, immunosuppression for autoimmune‐mediated subtypes, and corticosteroids for hypersensitivity‐type presentations. Finally, this LNP‐centric view will galvanize the optimization of the vaccine platform itself. The development of next‐generation ionizable lipids that are more biodegradable and less inflammatory is already underway and represents a promising path forward.

While vaccine‐associated myocarditis is a rare event—with most cases resolving favorably, though a subset may exhibit persistent cardiac imaging abnormalities warranting continued surveillance—and the benefits of vaccination overwhelmingly outweigh the risks, a clear understanding of its mechanism is essential. We acknowledge that the LNP‐centered model presented here represents one important pathogenic pathway among several that contribute to different clinical phenotypes. Spike protein‐mediated effects, autoimmune responses, and hypersensitivity reactions may each play a role in distinct patient subgroups. Elucidating these diverse pathophysiological pathways, including the LNP‐driven pathophysiology, will not only enhance the safety of current and future vaccines but will also solidify public trust in this revolutionary technology, better preparing us for health challenges to come.

## Funding

This work was supported by the National Research Foundation of Korea (NRF) grant funded by the Korea government (MSIT) (Grant RS‐2024‐00346434). This research was supported by Basic Science Research Program through the National Research Foundation of Korea (NRF) funded by the Ministry of Education (Grant 2020R1I1A1A01067800).

## Conflicts of Interest

The authors declare no conflicts of interest.

## Data Availability

The data that support the findings of this study are available from the corresponding author upon reasonable request.
